# Association between cesarean section and constipation in infants: the Japan Environment and Children’s Study (JECS)

**DOI:** 10.1186/s13104-018-3990-5

**Published:** 2018-12-12

**Authors:** Taketoshi Yoshida, Kenta Matsumura, Akiko Tsuchida, Kei Hamazaki, Hidekuni Inadera, Japan Environment and Children’s Study Group, Yukihiro Ohya, Reiko Kishi, Nobuo Yaegashi, Chisato Mori, Zentaro Yamagata, Takeo Nakayama, Masayuki Shima, Yasuaki Hirooka, Narufumi Suganuma, Koichi Kusuhara, Takahiko Katoh

**Affiliations:** 1 Division of Neonatology, Maternal and Perinatal Center, Toyama University Hospital, 2630 Sugitani, Toyama, 930-0194 Japan; 2 Toyama Regional Center for JECS, University of Toyama, Toyama, Japan; 3 Department of Public Health, Faculty of Medicine, University of Toyama, Toyama, Japan

**Keywords:** Birth cohort, Cesarean section, Constipation, Microbiota

## Abstract

**Objective:**

There have been increasing reports on the association between cesarean section (C-section) and the subsequent development of diseases in infants. C-section affects the diversity of microbiota in the infant’s gut. In the present study, we investigated the association between infants delivered by C-section and the development of constipation at 1 year old due to altered gut microbiota using data from the Japan Environment and Children’s Study (JECS).

**Results:**

This cohort study (n = 83,019) used data from JECS, an ongoing cohort study which began in January 2011. Data on bowel movement and potential confounding factors were recorded. A log-binomial regression model was used to estimate the risk of C-section, and the results were expressed as risk ratios and their respective 95% confidence intervals. Although infants delivered by C-section were of significantly younger gestational age and lesser birth weight than vaginally delivered infants, the frequency of bowel movements was almost similar between the two, independent of the mode of delivery. The prevalence of constipation in the entire infant was 1.37%. No significant differences were observed for C-section in crude and adjusted risk ratios for constipation.

## Introduction

During the last few decades, there have been increasing number of reports on the association between cesarean section (C-section) and the development of diseases later in childhood. C-section has been associated with several diseases in infants, such as asthma, gastroenteritis, inflammatory bowel disease, celiac disease, type 1 diabetes mellitus, and obesity [1–5]. Because infants born by C-section are not exposed to the mother’s vaginal and intestinal microbiota, the microbiota in these infants is similar to the mother’s skin flora [6]. In mice, the first exposure to microorganisms seems to be important for gut microbiota and priming of the regulatory immune system [7]. Thus, the mode of delivery may be a crucial factor for infants in terms of the development of the immune system and subsequent incidence of disease.

Functional gastrointestinal disorders, such as infantile colic, gastroesophageal reflux, and constipation, may be caused by abnormal intestinal flora [8]. Disruption of the intestinal microbiota is considered an etiological factor in pediatric functional constipation [9]. We therefore hypothesized that infants delivered by C-section could show increased morbidity to constipation due to altered gut microbiota.

In the present study, we investigated the association between infants delivered by C-section and the development of constipation using data from the Japan Environment and Children’s Study (JECS).

## Main text

### Methods

In this study, data were obtained from JECS, an ongoing birth cohort study that commenced in January 2011. JECS was designed to investigate mothers and children using surveys until their newborns reached the age of 13 years. It is scheduled to continue up to 2027 and is measuring the effect of environmental factors on children’s health. The detailed methodology has been previously reported [10].

Briefly, pregnant women from 15 regions in Japan were recruited for 3 years from 2011 to 2014. The JECS protocol was approved by the Review Board on Epidemiological Studies of the Ministry of the Environment and by the Ethics Committees of all participating institutions. JECS was conducted after obtaining written informed consent from all participants. A total of 103,062 pregnancies have participated in JECS (set jecs-an-20180131). After excluding multiple participation, multiple births, and miscarriages or still births, the remaining 92,790 women had singleton live births. Missing information about the mode of delivery or the infants’ defecation habit resulted in the exclusion of 9771 women; consequently, 83,019 participants were analyzed in the present study.

Follow-up was accomplished with self-administered questionnaires, which were completed at 1 month, 6 month and 1 year after birth. The questionnaires collected data related to the breastfeeding, stool condition, and confounding factors. Medical data were collected from the transcribed medical records and included gestational age, birth weight, mode of delivery and maternal age. The rate of exclusive breastfeeding was determined at 1 month after birth. Constipation was defined as chronic fecal retention characterized by defecation frequency of < 3 per week at 1 year old.

#### Statistical analyses

The correlation between C-section and constipation in infants was assessed. A log-binomial regression model was used to estimate the crude risk, confounding-adjusted risk, and 95% confidence interval (CI) for C-section-delivered infants. The following potential factors were assessed for confounding-adjusted risk: Adjusted for maternal age, marital status, body mass index before pregnancy, employment status, total income, education, maternal allergy, exercise and total calorie intake during pregnancy, smoking and alcohol consumption after 1 month delivery, previous delivery, gestational age, birth weight, sex of the neonate, congenital anomaly of the neonate, passive smoking and feeding at 1 month birth, and nursing at 6 months. All statistical analyses were performed using SAS version 9.4 (SAS Institute Inc., Cary, NC, USA).

### Results

Clinical characteristics of participants are shown in Table 1. C-section-delivered infants had younger gestational age and lesser birth weight than those of vaginally delivered infants (p < 0.001). The rate of exclusive breastfeeding was lower and maternal age was higher in the C-section group than in the vaginal delivery group (p < 0.001). The frequency of bowel movements is shown in Table 2. There was no difference between the groups at 1 year old. The prevalence of infant constipation in the entire cohort was 1.37%. Crude and adjusted risk ratios for constipation in C-section-delivered infants are shown in Table 3. No significant differences were observed between the two groups at 1 year old.

**Table 1:** Clinical characteristics of subjects

	Vaginal delivery	Cesarean delivery	p
N (male/female)	67,504 (34,643/32,861)	15,515 (7981/7534)	0.79
Gestational age, weeks	39.5 ± 1.3	38.2 ± 2.1	< 0.001
Birth weight, g	3060 ± 379	2869 ± 515	< 0.001
Missing, *n*	14		
Exclusively breast fed, *n* (%)	29,336 (43.5)	5499 (35.4)	< 0.001
Missing, *n*	726		
Maternal age, years	30.8 ± 4.9	32.5 ± 1.7	< 0.001
Missing, *n*	443		

Gestational age, Maternal age: mean And standard deviation

**Table 2:** Frequent bowel movement

	Vaginal delivery (n = 67, 504)	Cesarean delivery (n = 15,515)
	n (%)	n (%)
Frequency (week)		
0–2	940 (1.4)	198 (1.3)
3–6	7934 (11.8)	1737 (11.2)
7–14	49,567 (73.4)	11,359 (73.2)
≥ 15	9063 (13.4)	2221 (14.3)

**Table 3:** Crude and adjusted risk ratios for constipation

	Crude (n = 83,019)			Adjusted (n = 71,489)^a^		
				OR	(95% CI)	p value
	OR	(95% CI)	p value			
Cesarean delivery	1.09	(0.94, 1.28)	0.26	0.94	(0.79, 1.13)	0.51

*CI* confidence interval, *OR* odds ratio

^a^Adjusted for maternal age, marital status, body mass index before pregnancy, employment status, total income, education, maternal allergy, exercise and total calorie intake during pregnancy, smoking and alcohol consumption after 1 month delivery, previous delivery, gestational age, birth weight, sex of the neonate, congenital anomaly of the neonate, passive smoking and feeding at 1 month birth, and nursing at 6 months

### Discussion

In the present study, C-section was not associated with constipation in infants. This is the first report which addresses the direct association between cesarean section and constipation in infants. Because infants born by C-section have a less diverse gut flora [6, 7], we expected that they would tend to show constipation. However, the frequency of bowel movement was the same for both the groups (Table 2). Most instances of constipation in infants occurred at the time of transition from breastfeeding to consumption of infant formula [11]. Mothers who delivered by C-section had lower rates of exclusive breastfeeding than those who delivered vaginally (Table 1). Bowel movements were analyzed by excluding the mode of feeding (breastfeeding or infant formula) to demonstrate the effect of the mode of delivery.

Although there are several definitions of constipation, bowel movement of < 3 per week is acceptable in infants [12]. In the present study, the rate of constipation (< 3 week) was 1.37%, which is considerably lower than Western countries (Italy, 17.6% [11]; USA, 5% [13]). The prevalence of infants’ constipation has been reported range 0.05% to 0.3% in Thailand which is low rate like Japan [14]. The large variation in prevalence of constipation may be due to different country and food culture. This low rate of constipation in Japanese infants may have resulted in no differences between the groups in present study.

### Conclusion

This study showed that C-section has no effect on the frequency of bowel movement in infants at 1 year old.

## Limitations

Our study had several limitations. First, there were differences in the backgrounds of the two groups (Table 1). C-section-delivered infants were younger and had lesser birth weight than vaginally delivery infants, which may have resulted in differences in bowel maturation. However, Iacono et al. found that preterm birth is not associated with constipation [11]. Second, regarding the timing of analysis, there is a wide range of age with respect to the onset of constipation [12]. A diagnosis of constipation was made at 1 year of age in this study. Most studies evaluate the prevalence of constipation in children between 2 and 14 years of age [15–17]. The onset of constipation occurs within the first year of life in almost 50% of the affected children [18]. To investigate the effect of C-section on constipation, further studies will be required to determine the prevalence of constipation across different age groups.

## Appendix

Members of the JECS (principal investigator, Toshihiro Kawamoto) as of 2018: Yukihiro Ohya (National Center for Child Health and Development, Tokyo, Japan), Reiko Kishi (Hokkaido University, Sapporo, Japan), Nobuo Yaegashi (Tohoku University, Sendai, Japan), Koichi Hashimoto (Fukushima Medical University, Fukushima, Japan), Chisato Mori (Chiba University, Chiba, Japan), Shuichi Ito (Yokohama City University, Yokohama, Japan), Zentaro Yamagata (University of Yamanashi, Chuo, Japan), Hidekuni Inadera (University of Toyama, Toyama, Japan), Michihiro Kamijima (Nagoya City University, Nagoya, Japan), Takeo Nakayama (Kyoto University, Kyoto, Japan), Hiroyasu Iso (Osaka University, Suita, Japan), Masayuki Shima (Hyogo College of Medicine, Nishinomiya, Japan), Yasuaki Hirooka (Tottori University, Yonago, Japan), Narufumi Suganuma (Kochi University, Nankoku, Japan), Koichi Kusuhara (University of Occupational and Environmental Health, Kitakyushu, Japan), and Takahiko Katoh (Kumamoto University, Kumamoto, Japan).

## Abbreviations

JECS: Japan Environment and Children’s Study
C-section: cesarean section
